# Microsporidia dressing up: the spore polaroplast transport through the polar tube and transformation into the sporoplasm membrane

**DOI:** 10.1128/mbio.02749-23

**Published:** 2024-01-09

**Authors:** Qing Lv, Liuyi Hong, Lei Qi, Yuqing Chen, Zhengkai Xie, Hongjie Liao, Chunfeng Li, Tian Li, Xianzhi Meng, Jie Chen, Jialing Bao, Junhong Wei, Bing Han, Qingtao Shen, Louis M. Weiss, Zeyang Zhou, Mengxian Long, Guoqing Pan

**Affiliations:** 1 State Key Laboratory of Resource Insects, Southwest University, Chongqing, China; 2 Chongqing Key Laboratory of Microsporidia Infection and Control, Southwest University, Chongqing, China; 3 Biomedical Research Center for Structural Analysis, Shandong University, Jinan, Shandong, China; 4 Department of Pathogenic Biology, School of Basic Medical Sciences, Shandong University, Jinan, Shandong, China; 5 School of Life Science, Southern University of Science and Technology, Shenzhen, Guangdong, China; 6 Department of Pathology, Albert Einstein College of Medicine, New York, USA; 7 College of Life Sciences, Chongqing Normal University, Chongqing, China; University of Geneva, Geneva, Switzerland

**Keywords:** microsporidia, polar tube, polar filament, vesicle, polaroplast, sporoplasm membrane, transport

## Abstract

Microsporidia are obligate intracellular parasites that infect a wide variety of hosts including humans. Microsporidian spores possess a unique, highly specialized invasion apparatus involving the polar filament, polaroplast, and posterior vacuole. During spore germination, the polar filament is discharged out of the spore forming a hollow polar tube that transports the sporoplasm components including the nucleus into the host cell. Due to the complicated topological changes occurring in this process, the details of sporoplasm formation are not clear. Our data suggest that the limiting membrane of the nascent sporoplasm is formed by the polaroplast after microsporidian germination. Using electron microscopy and 1,1′-dioctadecyl-3,3,3′,3′ tetramethyl indocarbocyanine perchlorate staining, we describe that a large number of vesicles, nucleus, and other cytoplasm contents were transported out via the polar tube during spore germination, while the posterior vacuole and plasma membrane finally remained in the empty spore coat. Two *Nosema bombycis* sporoplasm surface proteins (NbTMP1 and NoboABCG1.1) were also found to localize in the region of the polaroplast and posterior vacuole in mature spores and in the discharged polar tube, which suggested that the polaroplast during transport through the polar tube became the limiting membrane of the sporoplasm. The analysis results of Golgi-tracker green and Golgi marker protein syntaxin 6 were also consistent with the model of the transported polaroplast derived from Golgi transformed into the nascent sporoplasm membrane.

**IMPORTANCE:**

Microsporidia, which are obligate intracellular pathogenic organisms, cause huge economic losses in agriculture and even threaten human health. The key to successful infection by the microsporidia is their unique invasion apparatus which includes the polar filament, polaroplast, and posterior vacuole. When the mature spore is activated to geminate, the polar filament uncoils and undergoes a rapid transition into the hollow polar tube that transports the sporoplasm components including the microsporidian nucleus into host cells. Details of the structural difference between the polar filament and polar tube, the process of cargo transport in extruded polar tube, and the formation of the sporoplasm membrane are still poorly understood. Herein, we verify that the polar filament evaginates to form the polar tube, which serves as a conduit for transporting the nucleus and other sporoplasm components. Furthermore, our results indicate that the transported polaroplast transforms into the sporoplasm membrane during spore germination. Our study provides new insights into the cargo transportation process of the polar tube and origin of the sporoplasm membrane, which provide important clarification of the microsporidian infection mechanism.

## INTRODUCTION

Microsporidia are a large group of important parasites in agriculture and human health (1, 2). *Nosema bombycis* was identified in 1857 as the pathogen of Pébrine in the silkworm *Bombyx mori* in Europe (3); since then, over 200 genera and 1,700 species of microsporidia have been identified (4–6). These enigmatic pathogens have fascinated investigators for more than 160 years because of their wide range of hosts, from invertebrates to vertebrates, as well as their unique and highly specialized invasion apparatus (7–11). Microsporidia not only cause huge economic losses to agricultural production (12) but also are responsible for human diseases. It was initially thought that human infection was restricted to immune-compromised individuals; however, it is now known that that microsporidiosis can also occur in immune-competent individuals (13, 14).

Microsporidia form characteristic infectious spores, which can survive outside host cells (1, 11). Mature spores have a unique infection apparatus for invasion, which includes the polar filament (also defined as the polar tube after spore germination), polaroplast, and posterior vacuole (PV) (11, 15, 16). In mature spores, the polar filament is tightly coiled and forms a spring-like structure (11, 17, 18), which is a solid structure comprising electron-dense alternating concentric rings in cross-sections (6, 19). In 2022, the macrostructure of the polar filament in *Encephalitozoon hellem* was characterized by cryogenic electron microscopy (Cryo-EM) and demonstrated that the polar filament had a multi-layer concentric circle structure and that many bumps with a diameter of 2.5 nm were present on the second layer of polar filament (20). The polaroplast is a system of membrane-limited cavities in the anterior part of the spore, which occupies one-third to one-half volume of the mature spore (11, 17). The compartments of the polaroplast are shaped like lamellae, chambers, or tubules delimited by approximately 5-nm-thick unit membranes (11, 21). The posterior polaroplast is close to the linear portion of the polar filament and may function to stabilize the linear portion. The roughly bowl-shaped PV is found at the posterior end of the mature spores, and the PV membrane is interlaced with the polar filament coils, suggesting that it may interact with the polar filament (6, 21, 22). In addition, the PV is believed to play an important role in spore germination, providing dynamic support for polar tube extension and cargo rapid passage (1, 22–24). An appropriate stimulus activates the spore germination, and the polar filament everts from the thinnest part of the mature spore forming a hollow tube (polar tube), like “reversing finger of a glove” (6, 11). During spore germination, the enlargement of polaroplast and the expansion of PV are thought to increase the internal turgor pressure of the spore to promote polar filament release and push the spore contents into the polar tube (3, 6, 11). The evagination mechanism of the polar filament and the detailed infection process of microsporidia have not been fully elucidated (14, 25–28).

The sporoplasm is indispensable for microsporidian infection and proliferation. Once the polar filament is released, the sporoplasm components, including the nucleus, are transported through the polar tube. The sporoplasm then appears as a droplet at the distal end of the polar tube and remains attached to the polar tube for several minutes (7, 13). Seven polar tube proteins (PTPs) have been identified from a variety of microsporidia (12, 25, 26, 29–34), and many of them appear to be involved in polar tube attachment and interaction with the host (6, 11, 12). The interaction mechanism of the polar tube and sporoplasm remains to be fully defined (1, 6, 35–37). PTP4 from *E. hellem* has been demonstrated to interact with sporoplasm surface protein 1, which may assist in the attachment of the sporoplasm to the tip of polar tube (6). It has also been supposed that the sporoplasm that is released from polar tube has a limiting membrane derived from polaroplast (11, 38–40). However, there is no conclusive evidence to confirm this hypothesis. In this study, we provide data on the transport of nucleus and sporoplasm components through the polar tube and that the transported polaroplast forms the novel sporoplasm membrane after spore germination.

## RESULTS

### Polar tube extension and cargo transportation

The mature spores of *N. bombycis* were placed on the transmission electron microscopy (TEM) grid, and then using 0.1 M KOH, spore germination was induced. By TEM observation, we found that a large number of polar tubes were released (red triangles, Fig. S1A). In addition, the average length of the polar tubes was about 150 µm (Fig. S1B), which was nearly 30–50 times the length of the spores. Discontinuous high electron density materials were observed in the polar tube (green triangles, Fig. S1C). Interestingly, we found that the tip of most of the polar tubes was curved hook structure (yellow triangles, Fig. S1C through E), which might assist sporoplasm adhesion with the eversion polar tube.

**Fig 1:**
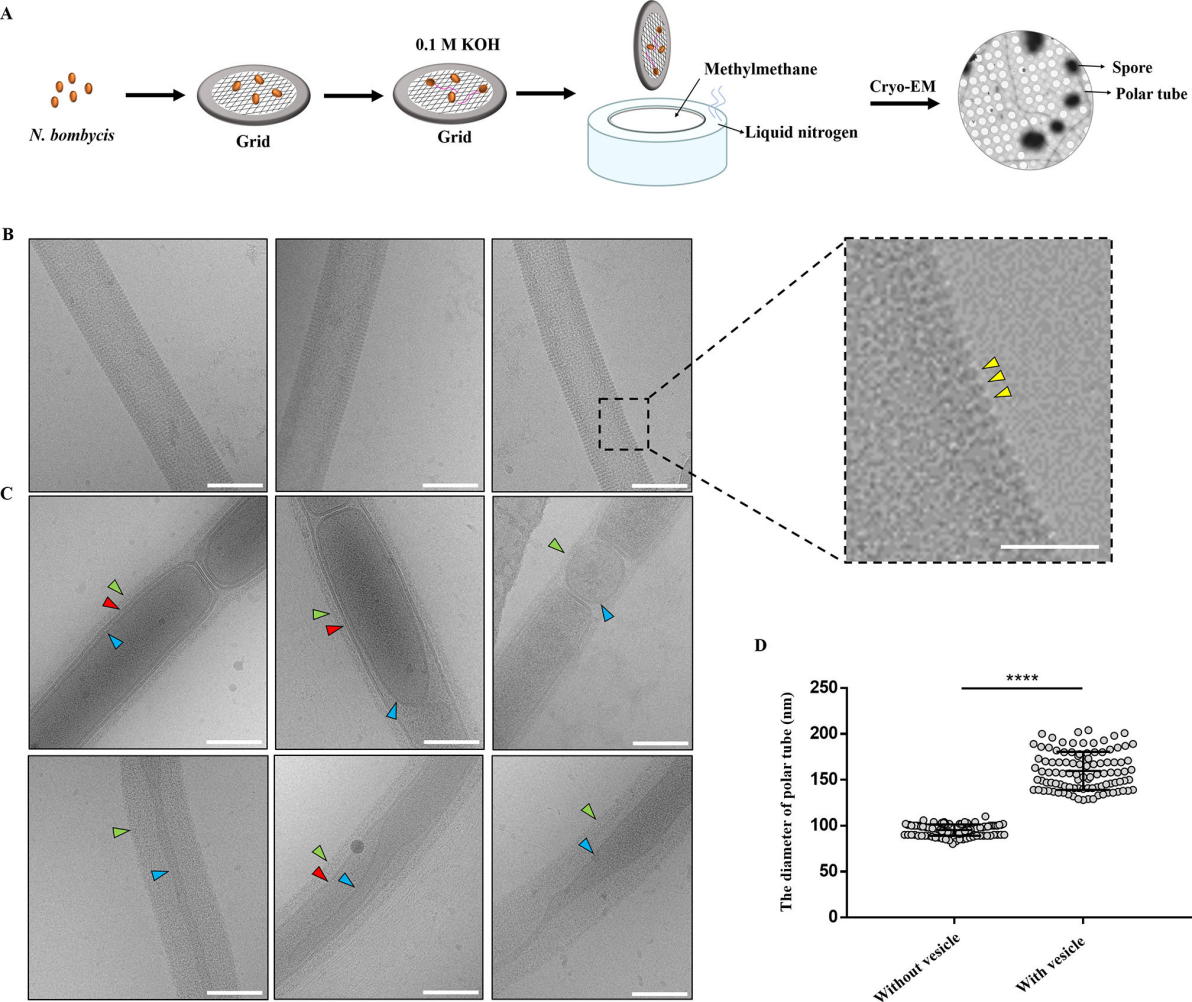
Cryo-EM analysis of the extruded polar tube of *N. bombycis*. (**A**) A schematic representation of the methodology for spore germination on the Cryo-EM grid. (**B**) Cryo-EM analysis of the structural characteristics of the polar tube without vesicles. Bar, 100 nm. The yellow triangles in the enlarged image pointed the bumps distributed on the surface of polar tubes. Bar, 50 nm. (**C**) Cryo-EM analysis of the structural characteristics of the polar tubes with vesicles. The green triangles pointed the fibrillar material on the surface of polar tubes. The blue triangles pointed the vesicle membrane inside the polar tube. The red triangles pointed the structure of membrane within membrane. Bar, 100 nm. (**D**) Comparison of the diameter of polar tube with and without vesicle. ****P < 0.0001 (*n* = 100 for each sample; unpaired Student’s *t*-test).

Spores were also placed on the Cryo-EM grid and germinated by 0.1 M KOH in order to characterize polar tube extrusion and cargo transportation *in situ* (Fig. 1A). Two types of polar tube were observed. One type was uniform with about 2-nm-long bumps distributed on its surface (yellow triangles, Fig. 1B). However, the surface of the other type of polar tube was no longer regular which was covered with relatively loose fibrillar material (green triangles, Fig. 1C). Obviously, a variety of vesicles distributed discontinuously in these polar tubes. These cargoes had different shapes, such as elliptic, long strips and circular. Most of them were monolayer membrane (blue triangles, Fig. 1C), whereas there was membrane-within-membrane structure in the polar tube (red triangles, Fig. 1C). The diameter of polar tubes without vesicle was only 80–110 nm, whereas the diameter of polar tubes containing vesicles varied greatly, ranging from 120 to 205 nm (Fig. 1D), indicating that the polar tube was an elastic tubular structure with the expansion capacity for cargo passage.

**Fig 2:**
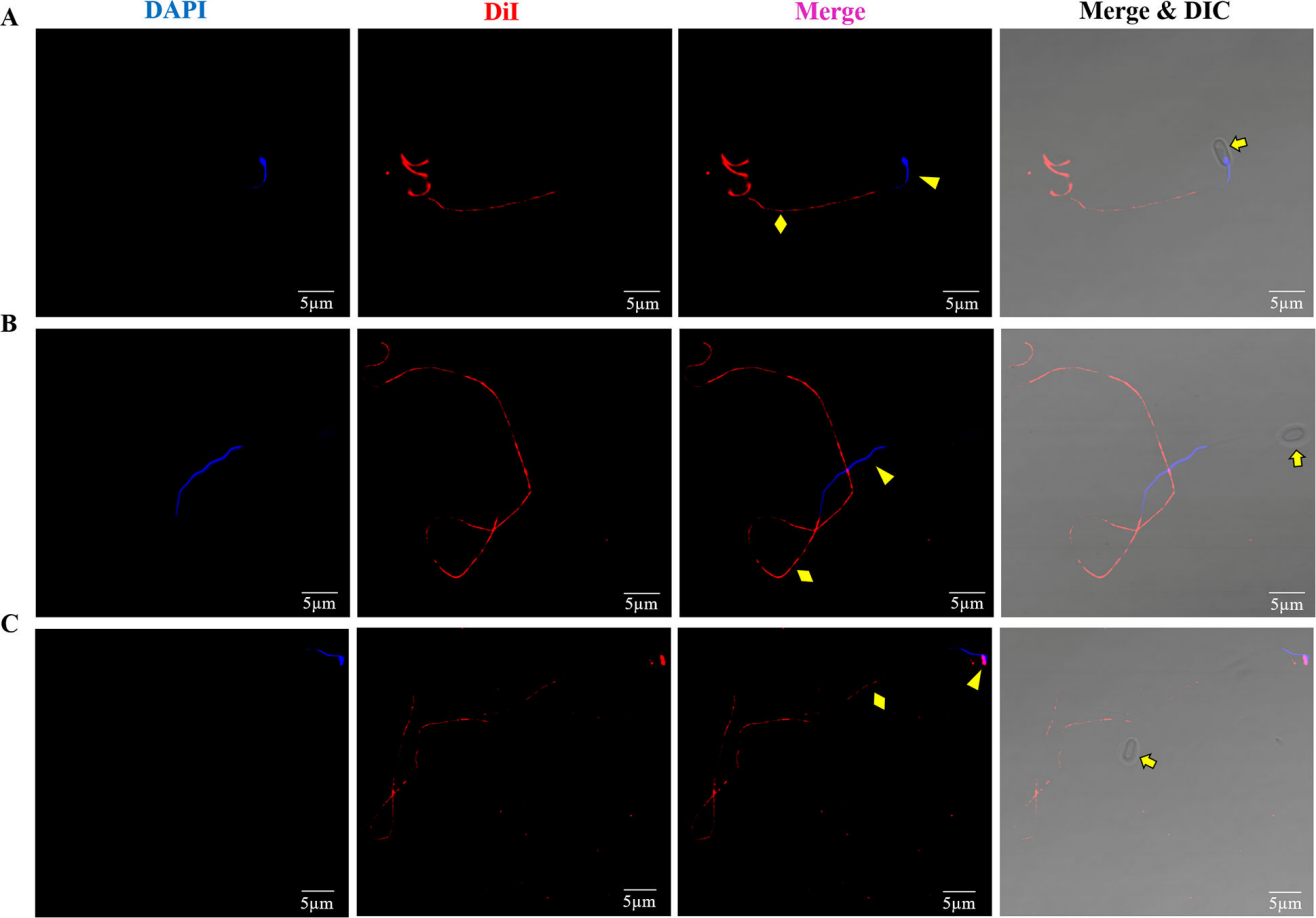
Fluorescent staining analysis of the cargo transport through the polar tube of *N. bombycis*. (**A**) One of the dikaryons had just entered the polar tube. (**B**) The nuclei were in the polar tube. (**C**) The nuclei were located at the tip of the polar tube. DAPI was used to label the nuclei (blue), and DiI was used to label the membrane material in the polar tube (red). The yellow arrows, triangles, and diamonds represented the spore, nuclei, and polar tube, respectively. Bar, 5 µm.

A lipophilic fluorescent dye 1,1′-dioctadecyl-3,3,3′,3′ tetramethyl indocarbocyanine perchlorate (DiI) and the nucleus dye DAPI were used to label the cargoes in the discharged polar tube. Spores were germinated using 0.1 M KOH for 3 minutes; then, the deformed nuclei were captured at a different location in the extruded polar tube (yellow triangles, Fig. 2A and B); and at the tip of the polar tube, they returned to the spherical shape (yellow triangle, Fig. 2C), which was consistent with findings reported in previous studies (21). In addition, no matter where the nuclei were located in the polar tube, the red fluorescence signal of DiI appeared discontinuously throughout the polar tubes (yellow diamonds, Fig. 2), implying that besides the nuclei, there were some discontinuous membrane structures in the polar tube, which was consistent with Cryo-EM observations (Fig. 1C). Moreover, the nuclei in the polar tube were not surrounded by DiI-labeled red fluorescence signal, whereas at the tip of the polar tube, there was a distinct co-location signal of DAPI-labeled nuclei and DiI-labeled membrane (Fig. 2C), suggesting that the nuclei of the sporoplasm enclosed a new membrane at the tip of the polar tube.

The extruded polar tube and sporoplasm were purified (Fig. S2A and B) according to the method reported in previous studies (32, 37). Quantitative lipidomic analysis of the extruded polar tube and sporoplasm found that the lipid composition of the polar tube and sporoplasm was very similar (Fig. S2C), which suggested that the lipid cargo transported thought that the polar tube was eventually transferred to the sporoplasm after spore germination.

### The spore polaroplast transport via polar tube during spore germination

During spore germination, the microsporidia undergo a transition from a highly compressed polar filament to a hollow polar tube that can transport infectious cargo. Therefore, we tried to determine whether these vesicle structures in the polar tube were also present in the polar filament. Using the methods reported in previous studies (32), we purified the polar filament fragments and labeled them with anti-NbPTP1 serum and DiI. The result showed the fragment of polar filaments that could be labeled green by NbPTP1 antibody but were not labeled by DiI (white arrows, Fig. S3A), which was suggested that the membrane structure that appeared in the polar tube was absent in the polar filament. In addition, there were continuous and relatively high electron density material in the central region of the polar filament; however, no vesicle was observed in the polar filament by Cryo-EM (Fig. S3B). Therefore, we speculated that these DiI-labeled membrane structure initially existed in mature spores and entered into the hollow polar tube during spore germination.

**Fig 3:**
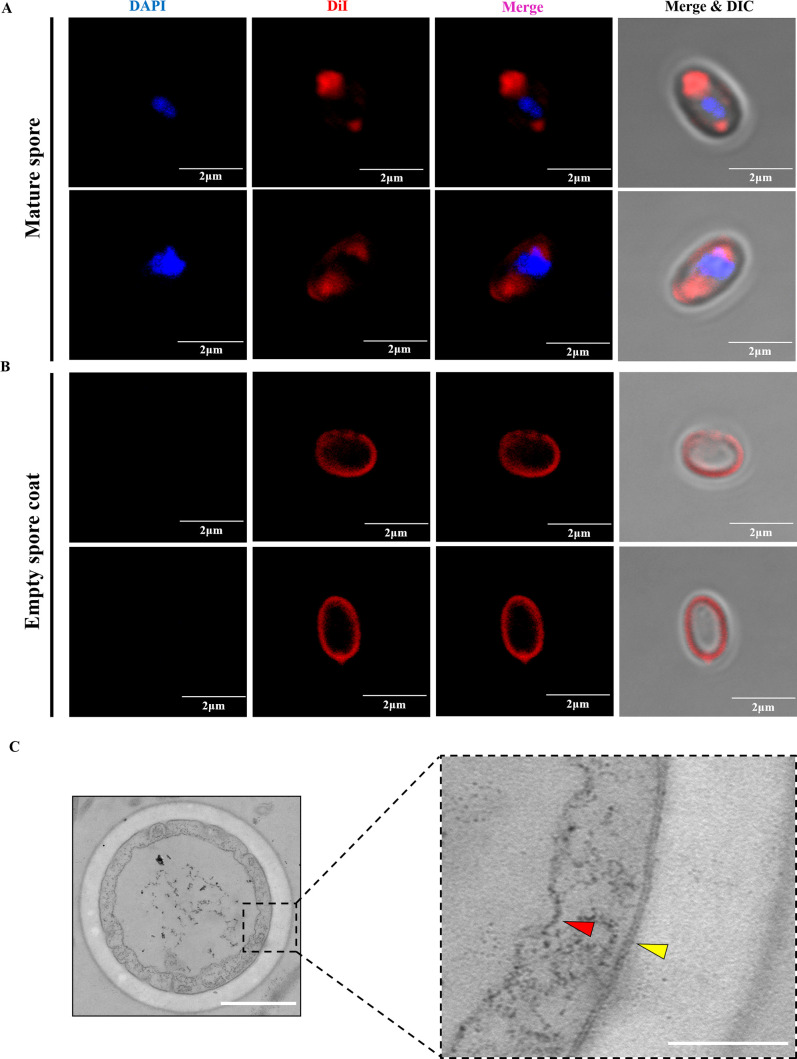
Analysis of the membrane structure in the mature spore and empty spore coat of *N. bombycis*. (**A**) Fluorescence analysis of the membrane structure in the mature spore by DiI staining. The nuclei were stained with DAPI (blue), and the membrane structure was stained with DiI (red). Bar, 2 µm. (**B**) Fluorescence analysis of the membrane structure in the empty spore coat by DiI staining. The nuclei were stained with DAPI (blue), and the membrane structure was stained with DiI (red). Bar, 2 µm. (**C**) TEM observation of the cross-section of the empty spore coat. Bar, 500 nm. The red and yellow triangles show the PV membrane and the plasma membrane, respectively, in the larger image of the black rectangle. Bar, 200 nm.

DiI was used to label mature spores, and red fluorescence signal was identified in the region of polaroplast and PV in the mature spores. In addition, the plasma membrane of the spores was also weakly stained (Fig. 3A). However, the DiI signal of the polaroplast and the PV region disappeared, and the signal near the plasma membrane region was significantly enhanced in the empty spore coats after spore germination (Fig. 3B). This indicated that parts of the membrane structure in the spores were transported out during spore germination and that the rest still remained in the empty spore coat.

Next, ungerminated and germinated spores of *N. bombycis* were sectioned and observed by TEM. In the mature spores, the polar filament (white arrows), the polaroplast (yellow dotted line), and the PV (white dotted line) were arranged in an orderly manner (Fig. S4A). During spore germination, the polar filament was released, and the PV gradually expanded to press the spore content into the polar tube (Fig. S4B). Eventually, the PV remained in the empty spore coat, which was consistent with previous studies (3, 11). Transverse section analysis of empty spore coats demonstrated that the following membranes existed in the empty spore coat: the plasma membrane (yellow triangle) and PV membrane (red triangle) (Fig. 3C). It was worth noting that the plasma membrane was a typical phospholipid bilayer structure, while the PV membrane appeared loose and irregular. The area circled by the yellow dotted line in mature spores had characteristics consistent with it being the polaroplast, with a large number of vesicles piled together (Fig. S4C), which were similar with the membrane structures in the polar tube (Fig. 1C). Based on these observations, we supposed that these DiI-labeled vesicle membrane structures in the polar tube were derived from the polaroplast membrane system.

### The process of the sporoplasm formation

**Fig 4:**
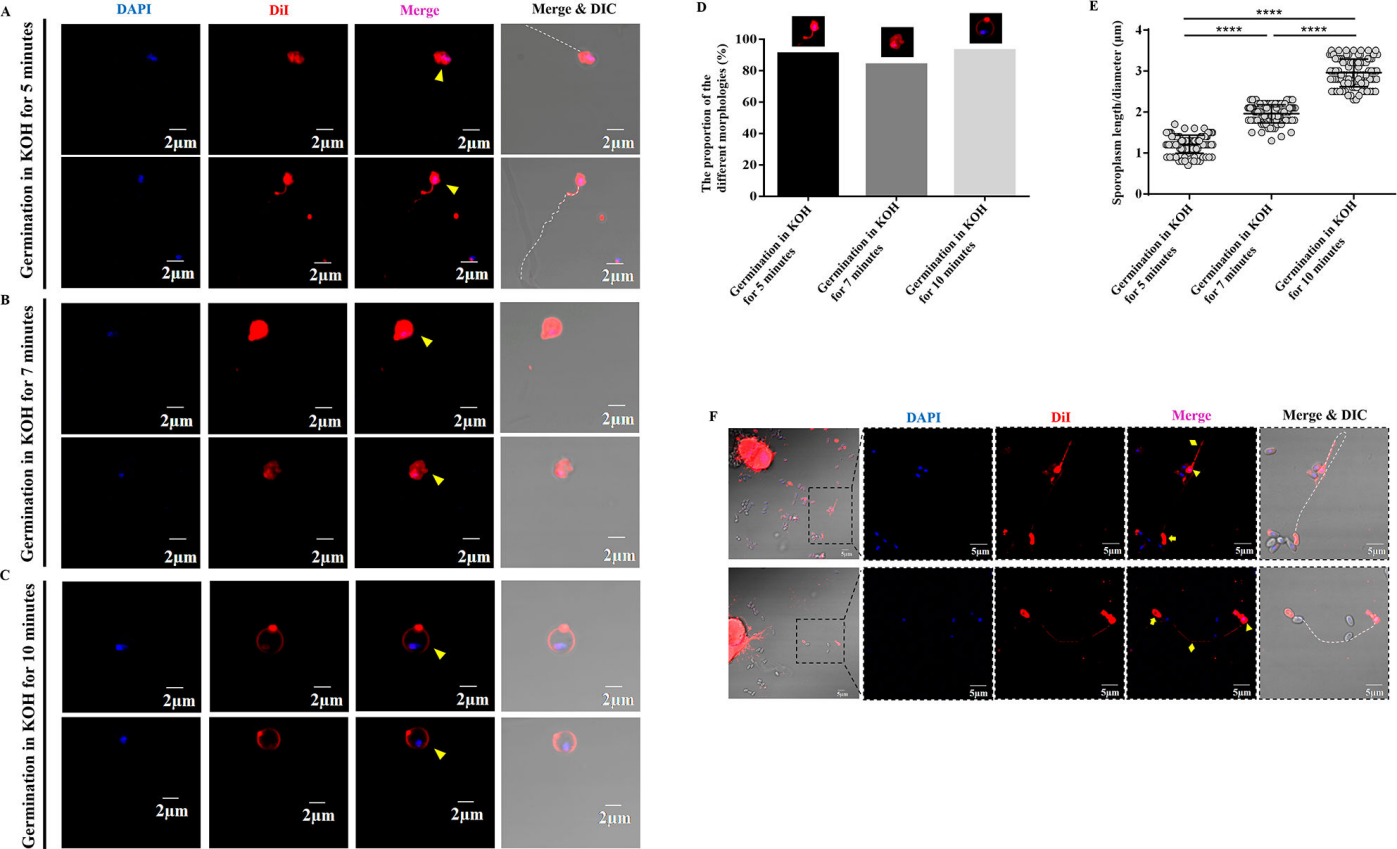
Analysis of the sporoplasm formation process. (**A**) Fluorescent staining analysis of mature spores treated in 0.1 M KOH for 5 minutes. The sporoplasms were located at the tip of the polar tube. Bar, 2 µm. (**B**) Fluorescent staining analysis of mature spores treated in 0.1 M KOH for 7 minutes. The sporoplasms with irregular shape fell off from the polar tube. Bar, 2 µm. (**C**) Fluorescent staining analysis of mature spores treated in 0.1 M KOH for 10 minutes. The free sporoplasms had a spherical shape. Bar, 2 µm. (**D**) The proportion of three different morphologies of sporoplasm in each treatment samples (*n* = 100 for each treatment). (**E**) The length or diameter of sporoplasms was calculated. ****P < 0.0001 (*n* = 100 for each treatment; unpaired Student’s *t*-test). (**F**) Fluorescence staining analysis of BmE cells co-cultured with *N. bombycis* spores. Bar, 5 µm. The nuclei were stained with DAPI (blue), and the membrane was stained with DiI (red). The yellow arrows, diamond, and triangles represent the empty spore coat, polar tube, and sporoplasm, respectively. The white dotted line indicates the polar tube.

We found that the plasma membrane remained in the empty spore coat after spore germination. Thus, where does the cell membrane of the sporoplasm come from? The sporoplasm formation process after *N. bombycis* spore germination was analyzed *in vitro*. After the spores were treated with 0.1 M KOH solution for 5, 7, and 10 minutes, the samples were stained with DAPI and DiI, respectively. After 5 minutes, we found that ~91% of the sporoplasms (yellow triangles) were attached to the tip of extruded polar tube (white dotted line), and the nuclei were wrapped in DiI-labeled membrane (Fig. 4A and D). At this time, the sporoplasm was irregular in shape, and the average length was 1.22 ± 0.02 µm (Fig. 4E). About 7 minutes later, ~84% of the sporoplasms were detached from the tip of polar tube (yellow triangles, Fig. 4B and D). In addition, the shape of sporoplasm was still irregular but tended to be round with almost 1.96 ± 0.02 µm in length (Fig. 4E). About 10 minutes later, ~93% of the sporoplasms became spherical, and the nuclei were located near the inner edge of the sporoplasm membrane (yellow triangles, Fig. 4C and D). The diameter of these sporoplasms increased significantly, with an average diameter of 2.97 ± 0.03 µm (Fig. 4E). The above data indicated that the nascent sporoplasm would firstly stick to the tip of polar tube and then gradually fell off from the polar tube. The cell membrane of the sporoplasm gradually became a circle enveloping the nuclei.

We co-cultured *N. bombycis* spores with silkworm *Bombyx mori* embryo (BmE) cells. After 24 hours, by staining the samples with DiI and DAPI, we found that the polar tube had been released from the mature spores, and some sporoplasms could be observed attaching to the tip of the polar tube. DiI-red fluorescence signals were present in the polar tube (yellow diamonds and white dotted line), the nascent sporoplasms (yellow triangles), and empty spore coats (yellow arrows) (Fig. 4F).

**Fig 5:**
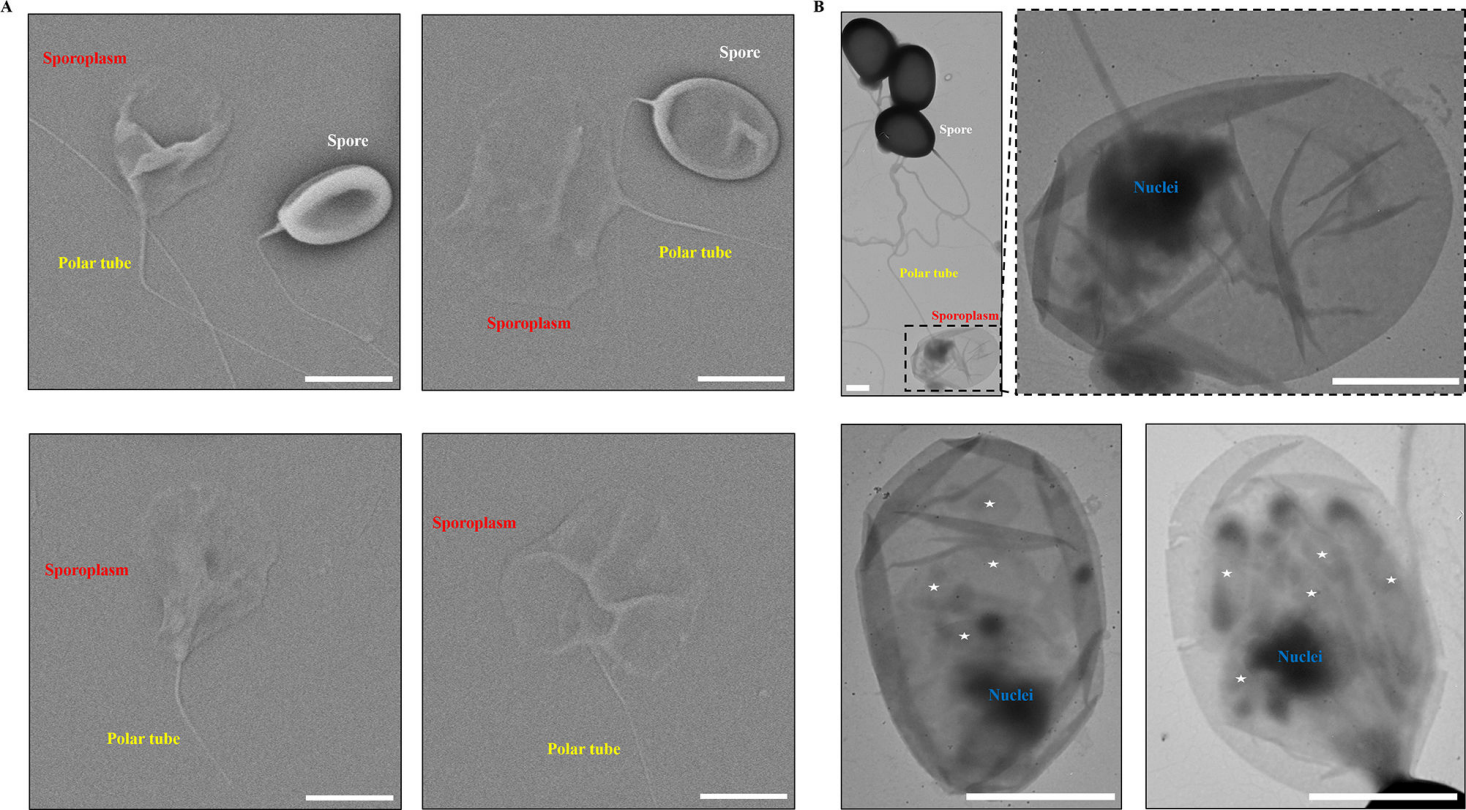
Electron microscopy analysis of the characteristics of *N. bombycis* sporoplasm. (**A**) SEM analysis of the sporoplasm. Bar, 2 µm. (**B**) TEM analysis of the sporoplasm. The asterisk represents the contents in the sporoplasm. Bar, 1 µm.

By scanning electron microscopy (SEM) and TEM analyses, the nascent sporoplasm expanded like a parachute, adhering to the polar tube (Fig. 5A). Sporoplasm consisted of a thin membrane with a low electron density and the nuclei with high electron density. There were other compartment-like structures besides the nuclei in the sporoplasm, which probably were other intracellular contents transported via the polar tube (white asterisks, Fig. 5B). We hypothesized that during spore germination, the polaroplast membrane system was transported to the tip of the polar tube and transformed into the cell membrane of the sporoplasm.

### The polaroplast forms the cell membrane of nascent sporoplasm during spore germination

**Fig 6:**
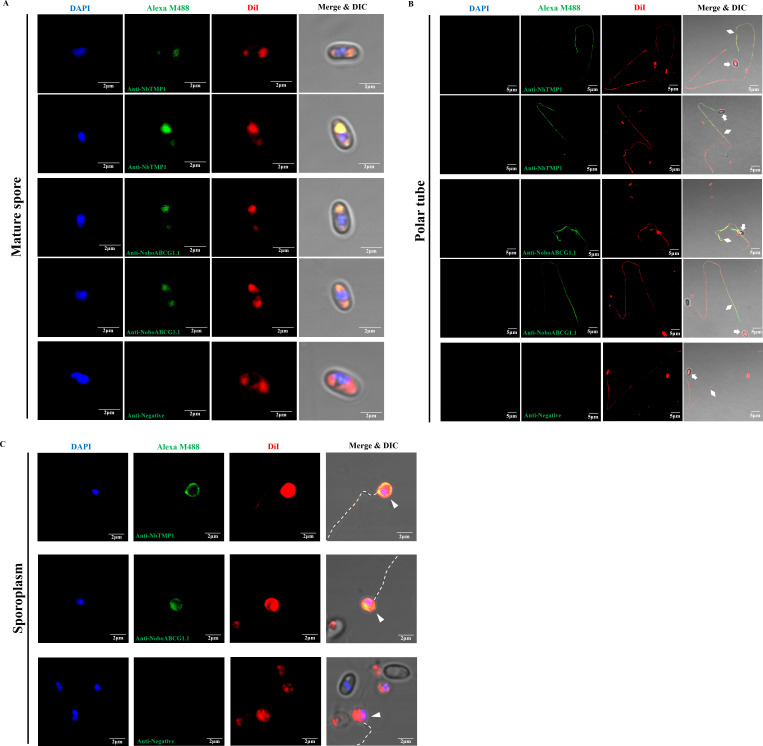
Immunofluorescence assay of NbTMP1 and NoboABCG1.1 localization in the mature spore, extruded polar tube, and sporoplasm of *N. bombycis*. (**A**) Immunofluorescence assay of NbTMP1 and NoboABCG1.1 localization in the mature spore. (**B**) Immunofluorescence assay of NbTMP1 and NoboABCG1.1 localization in the extruded polar tube. (**C**) Immunofluorescence assay of NbTMP1 and NoboABCG1.1 localization in the sporoplasm. The mature spore, polar tube, and sporoplasm samples were treated with mouse anti-NbTMP1 serum, mouse anti-NoboABCG1.1 serum, and negative serum, respectively. The nuclei were stained with DAPI (blue), and the membrane structure was stained with DiI (red). The white arrows, diamonds, and triangles indicate the spore empty coat, polar tube, and sporoplasm, respectively. The white dotted line indicates the extruded polar tube. Bar, 2 µm and 5 µm.

The *N. bombycis* proteins NbTMP1 and NoboABCG1.1 were identified as the surface proteins of sporoplasms (41, 42). It was found that both NbTMP1 and NoboABCG1.1 localized to the polaroplast region and PV region in mature spores (Fig. 6A). After spore germination, the discontinuous green fluorescence signal of NbTMP1 and NoboABCG1.1 antibody appeared in the extruded polar tube (Fig. 6B). Consistent with previous results (41, 42), NbTMP1 and NoboABCG1.1 were also detected on the cell membrane of sporoplasms (Fig. 6C). Interestingly, the fluorescence signal of NbTMP1 and NoboABCG1.1 antibody co-localized with the DiI signal. Considering that the PV still remained in the empty spore coat after spore germination, we speculated that the polaroplast membrane system was transported via the polar tube and transformed the cell membrane of nascent sporoplasms.

**Fig 7:**
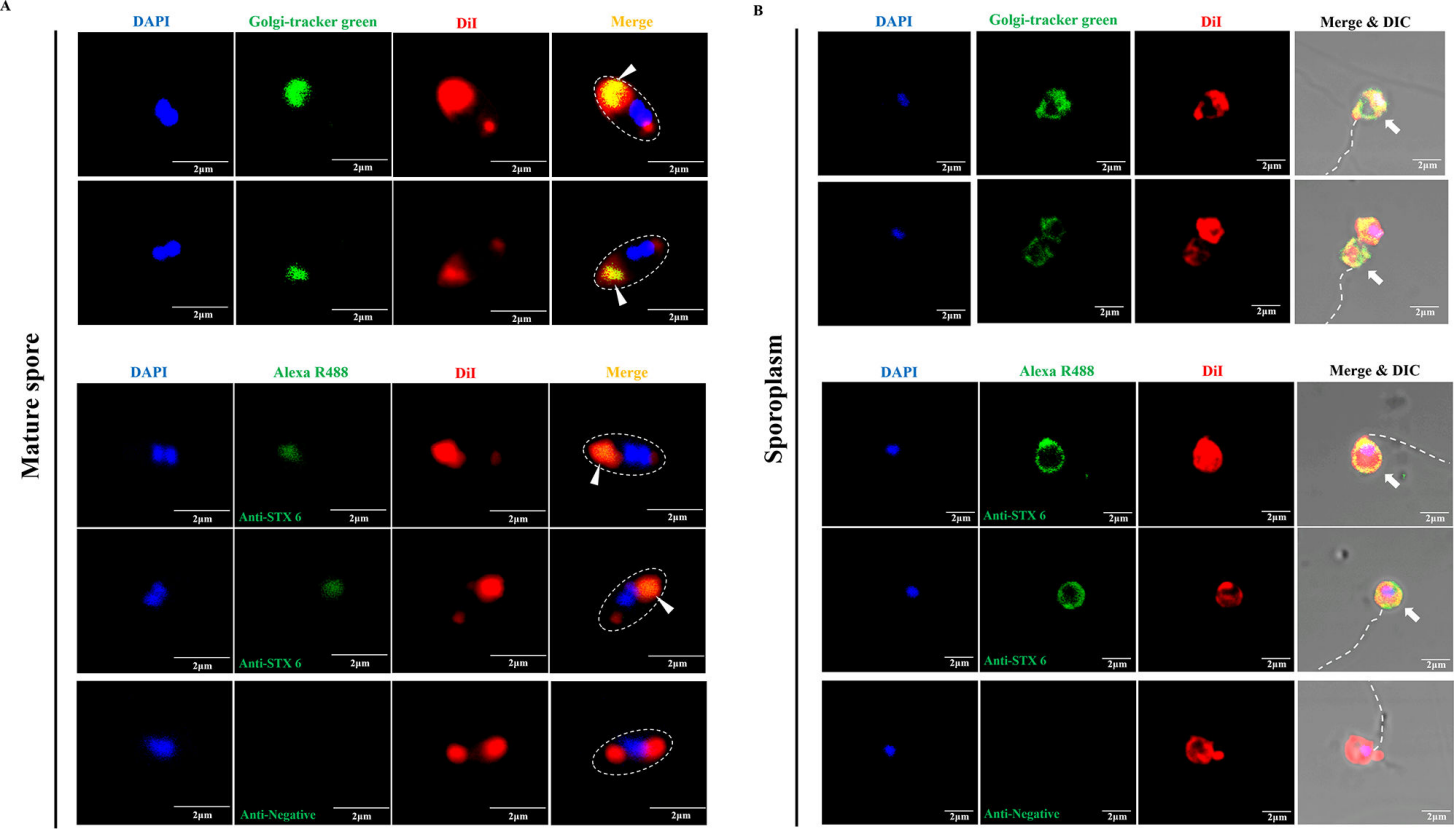
Analysis of the location of Golgi-tracker green and STX 6 in the mature spore and sporoplasm of *N. bombycis*. (**A**) Analysis of the location of Golgi-tracker green and STX 6 in the mature spore of *N. bombycis*. Golgi-tracker green was used for fluorescence staining analysis of the mature spore. Immunofluorescence analysis treated the mature spores with rabbit STX 6 polyclonal antibody and negative rabbit serum, respectively. (**B**) Analysis of the location of Golgi-tracker green and STX 6 in the sporoplasm of *N. bombycis*. Golgi-tracker green was used for fluorescence staining analysis of the sporoplasm. Immunofluorescence analysis treated the sporoplasms with rabbit STX 6 polyclonal antibody and negative rabbit serum, respectively. The nuclei were stained with DAPI (blue), and the membrane was stained with DiI (red). The white triangles and arrows indicate the polaroplast in the mature spores and sporoplasm, respectively. The white dotted line indicates the position of spore wall and the polar tube. Bar, 2 µm.

The origin of the polaroplast, which occupied a large volume in the mature spore, has not been clearly defined. Some studies have speculated that the polaroplast was probably of Golgi vesicle aggregation (11, 43, 44). Syntaxin 6 (STX 6) is a special protein marker for the Golgi complex (43, 45). Interestingly, we found a *N. bombycis* STX-like protein (GenBank no. EOB15057.1) that possesses sequence and structure homology to human STX 6 (GenBank no. CAG46671.1) (Fig. S5A and B). By immunoblot analysis, a polyclonal antibody against human STX 6 recognized a single band of approximately 25-kDa protein (consistent with the molecular weight of *N. bombycis* STX-like protein) in total spore proteins of *N. bombycis* (red arrow, Fig. S5C). Immunoelectron microscopy analysis further demonstrated that gold particles labeled by STX 6 antibody were distributed mainly at the polaroplast region of spores (Fig. S5D). Meanwhile, Golgi-tracker green (Beyotime, Shanghai, China) is another Golgi-specific marker. *N. bombycis* spores were labeled using Golgi-tracker green and human STX 6 antibody, respectively; it was found that both markers were localized to the spore polaroplast region which could co-localize with DiI signal (white triangles, Fig. 7A) and on the cell membrane of sporoplasms (white arrows, Fig. 7B). These results supported the hypothesis that the polaroplast derived from Golgi finally became the cell membrane of the nascent sporoplasm.

## DISCUSSION

All members of the microsporidia possess a unique, highly specialized invasion apparatus that includes the polar filament (called the polar tube after spore germination), polaroplast, and PV (1, 15, 16). Under appropriate environmental stimulation, the polar tube rapidly discharges out of the spore, pierces the host cell membrane, and acts as a conduit for sporoplasm passage into the new host cell (6, 11). As the highly specialized structure of the invasion apparatus, the characteristics of the polar tube are remarkable. The fine structure of polar tube from microsporidium *Anncaliia algerae* was analyzed by Cryo-EM in 2020 (23). The polar tube surface was pleated and covered with fine fibrillar material. In the polar tube, a variety of transported cargoes were observed, including cylinders, sacs, and vesicles filled with particulate material. In this study, we developed a microsporidian germination method on Cryo-EM grids to better analyze the native structure of extruded polar tubes. We found the polar tubes that were consistent with previous descriptions (23), whereas distinct with the reported *A. algerae* polar tube, we found another type of polar tube structure whose surface was no longer fibrillar material but neatly arranged with bumps of the same size like comb teeth, which might be the PTPs. This type of polar tube was filled with uniform electron density materials without any membrane structures in it, implying that no cargo transportation was occurring.

It has been hypothesized that the cell membrane of the sporoplasm is derived from the plasma membrane of mature spores (2, 6). However, electron microscopy examinations of *Glugea hertwigi* and *Spraguea lophii* demonstrated that the plasma membrane remained in the empty spore coat after the spore germination (40). Meanwhile, it is also hypothesized that the cell membrane of the sporoplasm originates from the polaroplast (6, 40), which is a system of membrane-limited cavities in the anterior part of the mature spores (11, 17, 46, 47). The expansion of polaroplast results in the disturbed arrangement of the membranous lamellae, thereby increasing turgor within the mature spore to promote the spore germination (6, 11, 48–50). N-phenyl-1-naphthylamine and chlorotetracycline both have affinity for membranes, and their fluorescent signals were found in the mature spore’s polaroplast and the sporoplasm membrane after spore germination, suggesting that the polaroplast might provide the new plasma membrane for discharged microsporidian sporoplasm (40). Therefore, the origin of the cell membrane of the sporoplasm is still unknown. Herein, two antibodies against sporoplasm surface protein of *N. bombycis* were found to also localize at the polaroplast and PV region in the mature spores, the extruded polar tube, and the cell membrane of the sporoplasm. Moreover, it was found that the PV remained in the empty spore coat and was not transported through the polar tube after spore germination. Therefore, it was mainly the polaroplast membrane system that transformed into the cell membrane of the nascent sporoplasm after being transported through the polar tube. The Golgi markers, Golgi-tracker green and STX 6 protein, were also found to localize at the polaroplast region and sporoplasm surface. Based on the above results, we believe that the cell membrane of sporoplasms is derived from the polaroplast which is originated from Golgi. Overall, these data support the hypothesis that sporoplasm membrane is from the polaroplast, but not the plasma membrane of mature spores.

The *N. bombycis* sporoplasm was ovate or spherical and about 1.5–2 µm in diameter (37). We found that the nascent sporoplasm was not a regular sphere attached to the tip of the polar tube but gradually returned to the round shape after falling off from the polar tube over time. There was a membrane fusion process during the process of sporoplasm shedding from the tip of the polar tube. This process, like blowing soap bubbles, may be very similar to the common process of cell refusion after fission (51–53). A hemi-fused, Ω-shaped structure has been observed for the first time in 2016 and is believed to play an important role in cell fusion. This structure was generated by opening and closing of fusion pore on the plasma membrane, and the process was rapidly accomplished (54–58). We also observed a convex punctate structure on the sporoplasm membrane, which might play a key role in membrane fusion process. Thus, we suspected that the stacked polaroplast membrane structures were transported through the polar tube and gradually unfold at the tip of the polar tube, finally enveloping the nucleus to form the sporoplasm.

**Fig 8:**
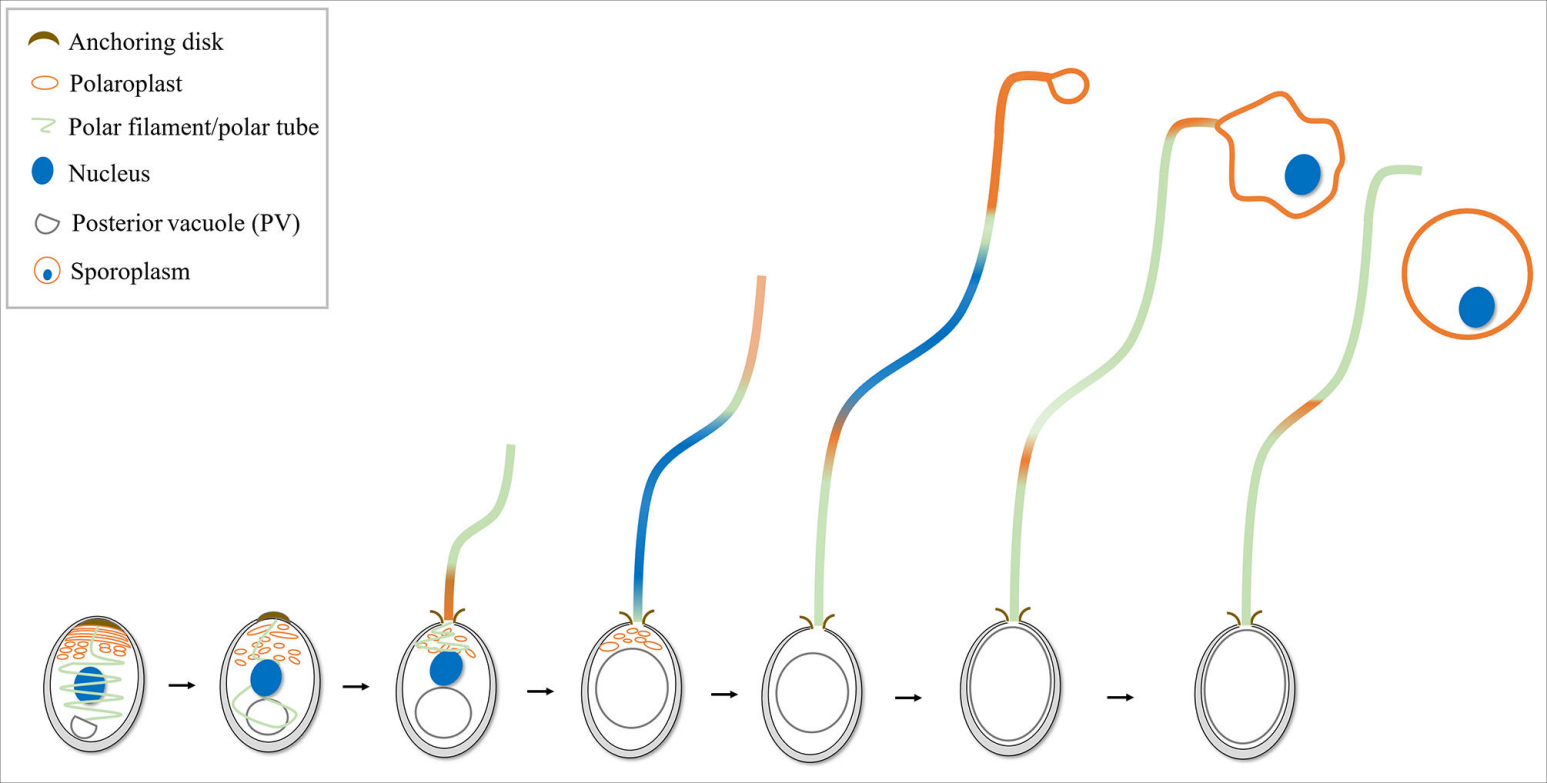
The process of microsporidian germination and sporoplasm formation. When microsporidian mature spore is appropriately stimulated, the PV becomes larger. Driven by the PV, the polar filament rapidly evaginates to transform the hollow polar tube, and the anterior part of polaroplast is injected into the polar tube. Then, the spore nucleus is elongated and transported through the polar tube. The polaroplast stays at the tip of the polar tube to form a bubble and encloses the nucleus. Finally, the sporoplasm falls off from the polar tube.

The data accumulated are basis for a model for the germination process of microsporidia cargo transport and sporoplasm formation (Fig. 8). When a microsporidian spore is appropriately stimulated, the PV becomes larger, and driven by the PV, the polar filament rapidly evaginates and the anterior polaroplast is injected into the polar tube. Then, the nucleus deformed and transported outside through the polar tube. The folded polaroplast stays at the tip of the polar tube and expands to form a bubble for enveloping the nucleus. Finally, the sporoplasm falls off from the polar tube. It is noteworthy that both the plasma membrane and PV remain in the empty spore coat after spore germination. However, the topology of the polar filament in the mature spore and the relationship between the polar filament and plasma membrane and PV remain to be fully determined.

## MATERIALS AND METHODS

### Cell culture

BmE cells were cultured in Grace medium supplemented with 10% fetal bovine serum (Gibco, Thermo Fisher Scientific, Shanghai, China) at 28°C.

### Preparation and purification of microsporidian spores

Fourth-instar silkworms were infected with *N. bombycis* isolate CQ1 spores (1 × 10^4^ spores per larva). The infected silkworm pupa was ground with a small amount of ddH_2_O, until there was no obvious flocculent. The material was filtered through two layers of nylon mesh and cleaned repeatedly until the supernatant was clarified by centrifugation at 3,000 rpm for 10 minutes. Finally, the pellet was evenly suspended with ddH_2_O and added to 90% Percoll (Sigma-Aldrich, St. Louis, USA) for centrifugation at 13,000 rpm for 20 minutes. The pellet was collected to be the mature spores, which was stored at 4°C.

### Microsporidia co-culture with host cells

*N. bombycis* spores were added to a round glass coverslip containing about 90% confluence BmE cells in the 12-well plate, and a final spore ratio was 20:1 (spores:cell) (59). After co-culture for 24 hours, the coverslips in the 12-well plate were washed gently with PBS (136 mM NaCl, 8 mM Na_2_HPO_4_, 2 mM KH_2_PO_4_, 2.6 mM KCl) and used as the sample for fluorescence staining.

### Fluorescent staining analysis

The samples used for fluorescent staining analysis experiments included four groups. The mature spores were purified according to the above methods, the germinated spore samples were obtained by treating mature spores in 0.1 M KOH, co-culture samples of microsporidia and host cells were obtained according to the above methods, and the purified sporoplasm samples *in vitro* were produced using previously published methods (37). For mature spores, 20 mg/mL protease K was used to treat the spore wall protein for 30 minutes at 58°C and centrifuged at 3,000 rpm for 5 minutes; the pellet was washed with ddH_2_O for several times. PMSF (Phenylmethylsulfonyl fluoride, 100 mM) was added to inactivate the protease K, and then, the spores were treated with 0.5% Triton X-100 for 30 minutes at room temperature to facilitate better entry of the dye. Mature spores and germinated spores were ﬁxed with 4% (wt/vol) formaldehyde at room temperature for 25 minutes, and to observe the sporoplasm, the cell samples were fixed with 2.5% (vol/vol) glutaraldehyde for 25 minutes. Samples were stained by DiI (Thermo Fisher Scientific, Shanghai, China) for 15 minutes and with Golgi-tracker green (Beyotime, Shanghai, China) for 15 minutes. After washing, samples were stained with DAPI (Thermo Fisher Scientific, Shanghai, China) for 15 minutes. Finally, all the above samples were examined by the Olympus FV1200 laser confocal microscope (Olympus, Tokyo, Japan).

### Immunofluorescence assay

The purified polar filament and polar tube fragments *in vitro* were prepared by previously published methods (32). Mature spores were treated using the 20 mg/mL protease K for 30 minutes at 58°C and 0.5% Triton X-100 for 30 minutes at room temperature to facilitate better entry of the antibody. Mature spore samples, germinated spores with 0.1 M KOH samples, and the purified polar filament and polar tube samples were ﬁxed with 4% (wt/vol) formaldehyde at room temperature for 25 minutes and fixed with 2.5% (vol/vol) glutaraldehyde for 25 minutes. Then, all samples were blocked with 10% (vol/vol) non-speciﬁc goat serum together with 5% (wt/vol) bovine serum albumin (BSA) in PBST at room temperature for 1 hour. Samples were respectively incubated with anti-rabbit NbPTP1 (1:200 dilution), anti-mouse NbTMP1 (1:200 dilution), anti-mouse NoboABCG1.1 (1:200 dilution), and STX 6 polyclonal antibody (1:500 dilution) (ImmunoWay, Jiangsu, China) at room temperature for 1 hour. After being washed three times, samples were respectively incubated with Alexa Fluor 488 conjugate goat anti-rabbit IgG (1:1,000 dilution; Thermo Fisher Scientific, Shanghai, China) and Alexa Fluor 488 conjugate goat anti-mouse IgG (1:1,000 dilution; Thermo Fisher Scientific, Shanghai, China) for 1 hour at room temperature. After being washed for three times, the samples were stained with DiI for 15 minutes. Following washing three times, DAPI was used to stain the samples for 15 minutes. Finally, all the above samples were examined by the Olympus FV1200 laser confocal microscope (Olympus, Tokyo, Japan).

### Scanning electron microscopy

Germinated spore samples were fixed with 2.5% glutaraldehyde (Solarbio, Beijing, China) overnight at 4°C. Then, samples were fixed with 1% osmic acid (Ted Pella, Shanghai, China) for 1 hour and were dehydrated using a graded series of ethanol (30%, 40%, 50%, 60%, 70%, 80%, and 90%) for 10 minutes each treatment and two times 100% ethanol for 15 minutes. Following, the samples were dehydrated using tert-butyl alcohol: acetonitrile (2:1 and 1:1), followed by absolute acetonitrile for 10 minutes each treatment. Finally, the samples were observed with the scanning electron microscope (Phenom-World BV, Eindhoven, Netherlands).

### Transmission electron microscopy

*N. bombycis* spores (1 × 10^5^ spores/mL) were placed on nickel grids (Quantifoil, Beijing, China) for 10 minutes and germinated in 0.1 M KOH for 15 minutes at 30°C. Ultrathin sections (70 nm) of mature spores and germinated spores with 0.1 M KOH were prepared as previously described (60) and placed on nickel grids (Quantifoil, Beijing, China). All samples were washed three times by ddH_2_O at room temperature. Then, the samples were stained in 3% uranyl acetate (Zhongjingkeyi Technology Co., Ltd., Beijing, China), followed by lead citrate (Zhongjingkeyi Technology Co., Ltd., Beijing, China). Samples were observed using the Hitachi HT7800 electron microscope (Hitachi, Tokyo, Japan) at 80 kV.

### Immunoelectron microscopy

Ultrathin sections (70 nm) of mature spores were prepared as previously described (60) and placed on nickel grids (Quantifoil, Beijing, China). After blocking with 10% (vol/vol) non-specific goat serum together with 5% (wt/vol) BSA in PBST at room temperature for 1 hour, the grids were incubated with STX 6 polyclonal antibody (1:50 dilution; ImmunoWay, Jiangsu, China) or the negative rabbit antiserum at room temperature for 1 hour. The grids were then incubated with gold-conjugated anti-rabbit IgG (1:30 dilution; Sigma-Aldrich, St. Louis, USA). Then, the samples were stained in 3% (wt/vol) uranyl acetate (Zhongjingkeyi Technology Co., Ltd., Beijing, China), followed by 1% (wt/vol) lead citrate (Zhongjingkeyi Technology Co., Ltd., Beijing, China). Samples were observed using the Hitachi HT7800 electron microscope (Hitachi, Tokyo, Japan) at 80 kV.

### Cryogenic-electron microscopy

*N. bombycis* spores (1 × 10^8^ spores/mL) were placed on grids (Cu R2/1, 200 mesh) (Quantifoil Micro Tools GmbH, Jena, Germany) for 5 minutes and germinated in 0.1 M KOH for 15 minutes at 30°C. In addition, in order to observe the structure of the polar filament, we purified the polar filament fragments using the previously reported method (32) and then placed the polar filaments sample on grids for 5 minutes. Cryo-EM grids were rapidly prepared by plunge freezing in liquid ethane on a Vitrobot Mark IV (Thermo Fisher Scientific, USA). The conditions were set as follows: blot time was 2 s; blot force was −2. Then, samples were imaged on a 300-kV Titan krios G3i transmission electron microscope (Thermo Fisher Scientific, USA) using a Bioquantum K3 detector (Gatan) with a magnification of 81,000× and the defocus at −3 µm.

### Quantitative lipidomics

The extruded polar tubes and sporoplasms were analyzed according to the methods reported in previous studies (32, 37). Then, the polar tube and sporoplasm preparations were pretreated by methyl tert-butyl ether and separated by UHPLC Nexera LC-30A ultra-performance liquid chromatography. Electrospray ionization positive and negative ion modes were used for detection, respectively. The samples were separated by UHPLC and analyzed by mass spectrometry with Q Exactive series mass spectrometer (Thermo Fisher Scientific, Shanghai, China). The mass charge ratios of lipid molecules and lipid fragments were collected by the following method: 10 fragment maps were collected after each full scan (MS2 scan, HCD). The MS1 has a resolution of 70,000 at m/z 200, and the MS2 has a resolution of 17,500 at m/z 200. LipidSearch was used to carry out peak recognition, peak extraction, and lipid identification (secondary identification) of lipid molecules and internal standard lipid molecules. The parameters used for the analysis are as follows: precursor tolerance, 5 ppm; product tolerance, 5 ppm; product ion threshold, 5%. This analysis was completed in Shanghai Applied Protein Technology Co., Ltd. (Shanghai, China).

### Western blot

First, the total spore protein of mature spores was extracted (59). Concisely, mature spores (1 × 10^9^ spores/mL) were disrupted for 5 minutes for three times with 0.4-g acid-washed glass beads (0.2 g 212–300 μm and 0.2 g 425–600 μm; Sigma-Aldrich, St. Louis, USA) in 500-µL buffer PBS, pH 7.4, containing PMSF by Bioprep-24 Homogenizer (Beijing, China) and then centrifuged at 12,000 rpm for 5 minutes at 4°C. The supernatant was collected as the spore total protein samples. For immunoblotting analysis, 10-µg protein samples were subjected to SDS-PAGE, and the gel was transferred to the PVDF membrane (Roche, Shanghai, China). After blocking in 5% skim milk diluted in TBST (150 mM NaCl, 20 mM Tris-HCl, 0.05% Tween-20), the membrane was incubated with STX 6 polyclonal antibody (diluted 1:1,000 in blocking solution, ImmunoWay, Jiangsu, China) for 1.5 hours at 37°C. The negative rabbit antiserum was used as a negative control. Then, the membrane was washed and incubated with goat anti-rabbit IgG (1:8,000 dilution; Sigma-Aldrich, St. Louis, USA) for 1 hour at 37°C. Washing was performed three times with TBST, and the blot was developed with ECL Western blot detection kit (Thermo Fisher Scientific, Shanghai, China).

### Sequence analysis

The human STX 6 (GenBank no. CAG46671.1) and *N. bombycis* STX-like protein (no. EOB15057.1) sequences were obtained from the NCBI database. Multiple sequence alignments were generated with the ClustalX 1.83. The functional domain and transmembrane domain were predicted by SMART (http://smart.embl-heidelberg.de/) and Uniprot (https://www.uniprot.org/), respectively. The protein structure model was predicted by AlphaFold2 server (https://colab.research.google.com/github/sokrypton/ColabFold/blob/main/AlphaFold2.ipynb). The protein structural alignment was visualized by Chimera1.16.

### Statistical analysis

Fifty extruded polar tubes were randomly selected to measure the length of polar tubes of *N. bombycis* by ImageJ. In order to calculate the occurrence frequency of hook’s polar tubes, 100 polar tubes were randomly photographed by TEM, and the polar tube with and without hooks were manually counted. One hundred polar tubes with vesicles and without vesicles observed by Cryo-EM were randomly selected to measure the diameters of polar tube using ImageJ software. At least 20 fields of confocal microscopy observation with more than 100 germinated spores were used to randomly capture nuclei at different locations in the polar tube during spore germination. One hundred sporoplasms with typical characteristics in each group were randomly photographed to measure the length and the diameter of these sporoplasms using ImageJ software. GraphPad Prism software was used for all statistical analyses. In all analyses, the two-tailed unpaired Student’s *t*-test was used to compare the difference between two groups. *P* values are reported in the figure legends.

## Data Availability

All other relevant data are within the manuscript and its supplementary file.

## SUPPLEMENTAL MATERIAL

Fig. S1 mbio.02749-23-s0001.tif TEM analysis of the extruded polar tube of *N. bombycis*.

Fig. S2 mbio.02749-23-s0002.tif Purification of the polar tube and sporoplasm from *N. bombycis* and quantitative lipidomic analysis.

Fig. S3 mbio.02749-23-s0003.tif Structure characteristics of the polar filament in *N. bombycis*.

Fig. S4 mbio.02749-23-s0004.tif TEM analysis of the ungerminated and germinated spores of *N. bombycis*.

Fig. S5 mbio.02749-23-s0005.tif Characterization of the STX-like protein in *N. bombycis*.

Supplemental Information mbio.02749-23-s0006.docx Legends for Fig. S1 to S5.

ASM does not own the copyrights to Supplemental Material that may be linked to, or accessed through, an article. The authors have granted ASM a non-exclusive, world-wide license to publish the Supplemental Material files. Please contact the corresponding author directly for reuse.
