# Supplementary figures and images for: Microsporidia dressing up: the spore polaroplast transport through the polar tube and transformation into the sporoplasm membrane
Source: mBio. 2024 Jan 9;15(2):e02749-23. doi: 10.1128/mbio.02749-23 (PMC10865828; doi:10.1128/mbio.02749-23)

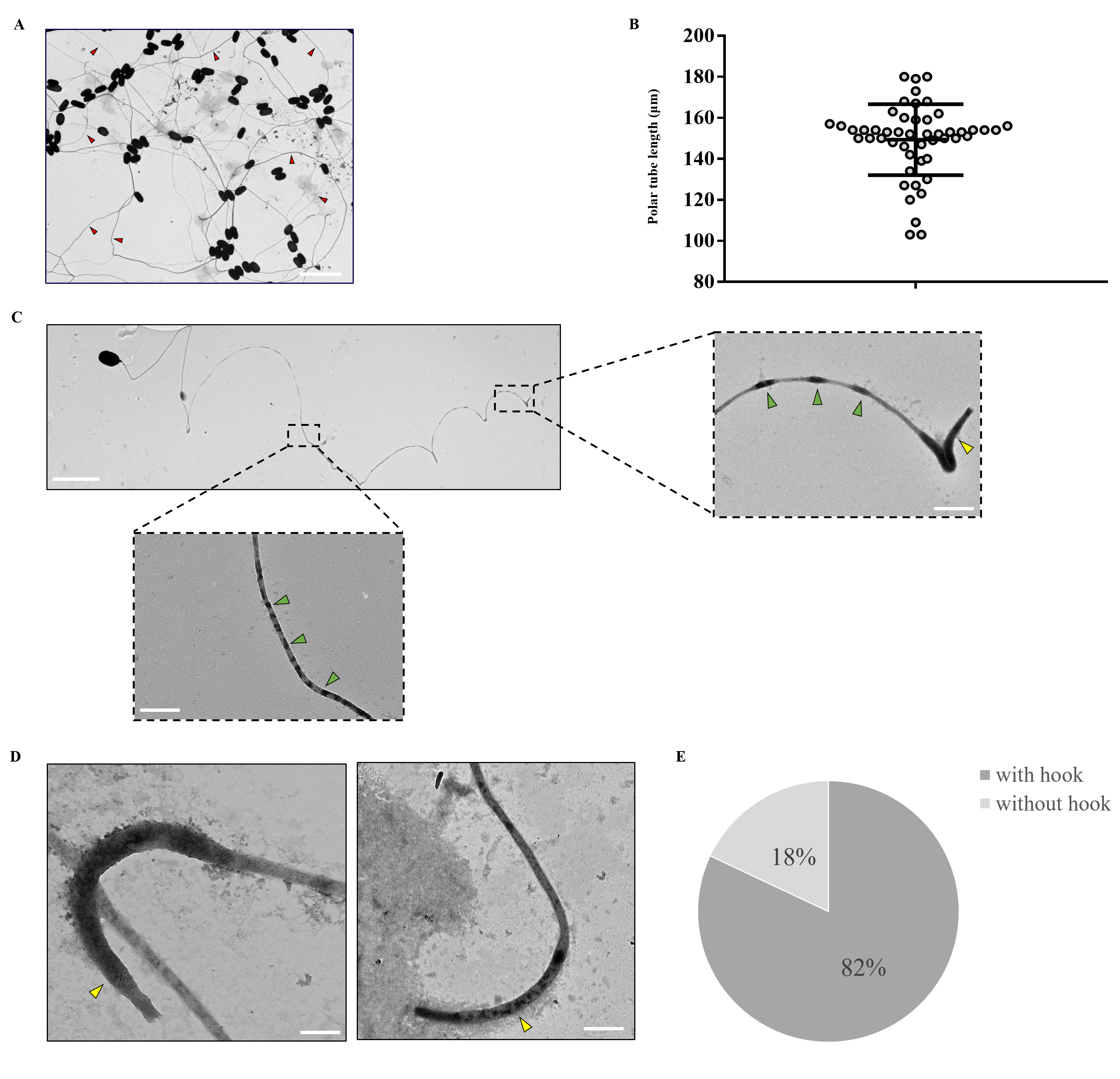

Supplement: Fig. S1 — TEM analysis of the extruded polar tube of N. bombycis. [file mbio.02749-23-s0001.tif]

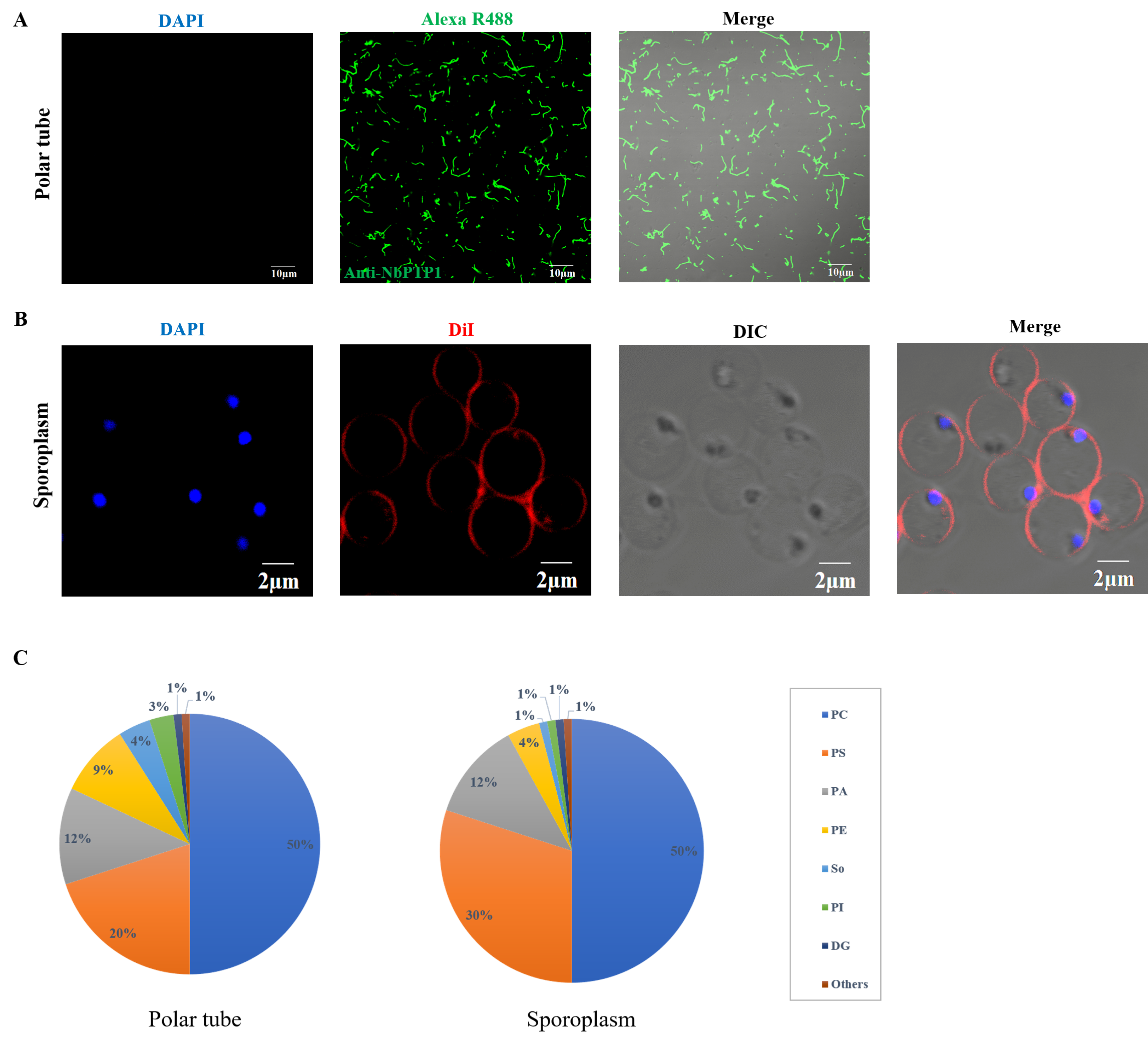

Supplement: Fig. S2 — Purification of the polar tube and sporoplasm from N. bombycis and quantitative lipidomic analysis. [file mbio.02749-23-s0002.tif]

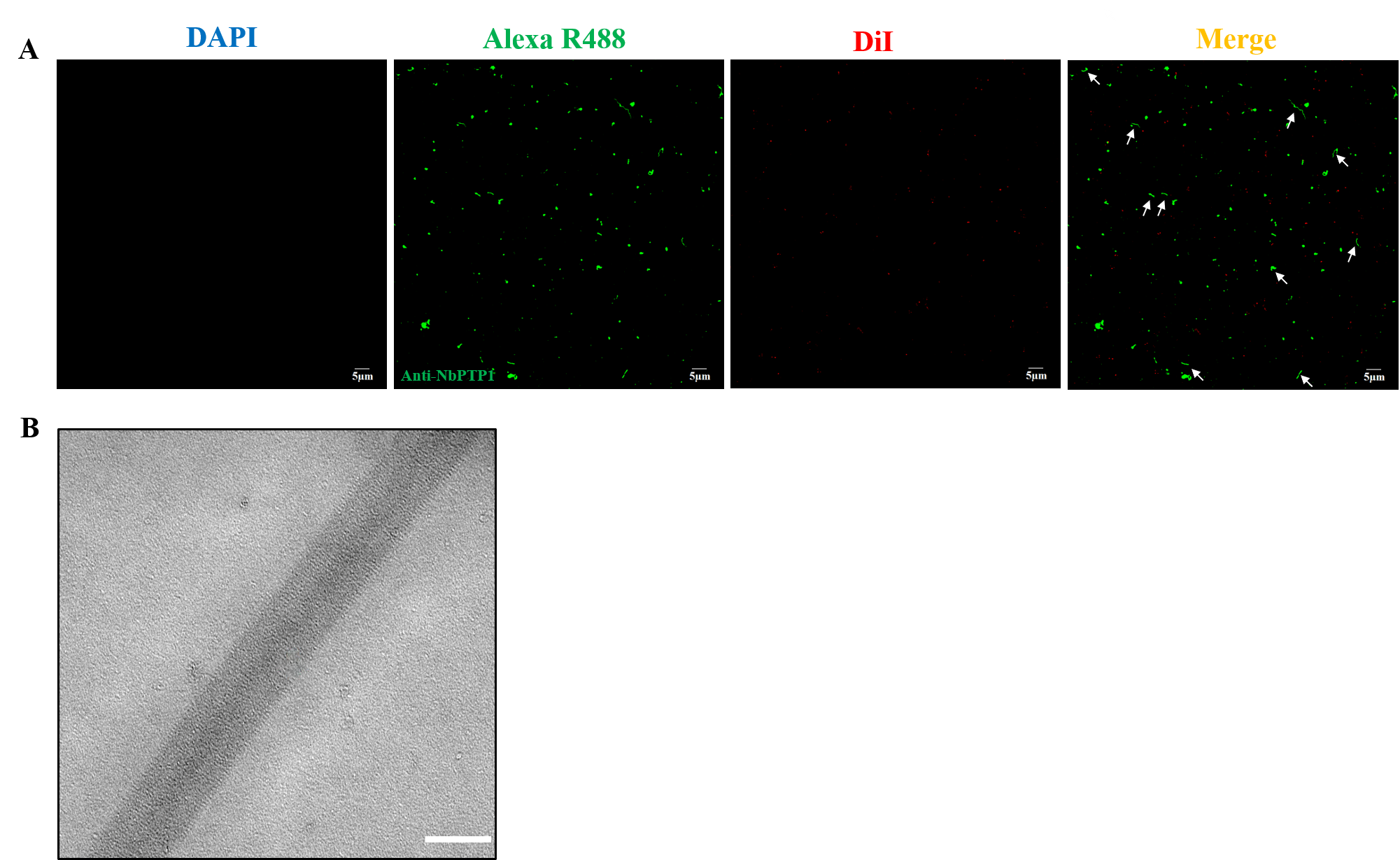

Supplement: Fig. S3 — Structure characteristics of the polar filament in N. bombycis. [file mbio.02749-23-s0003.tif]

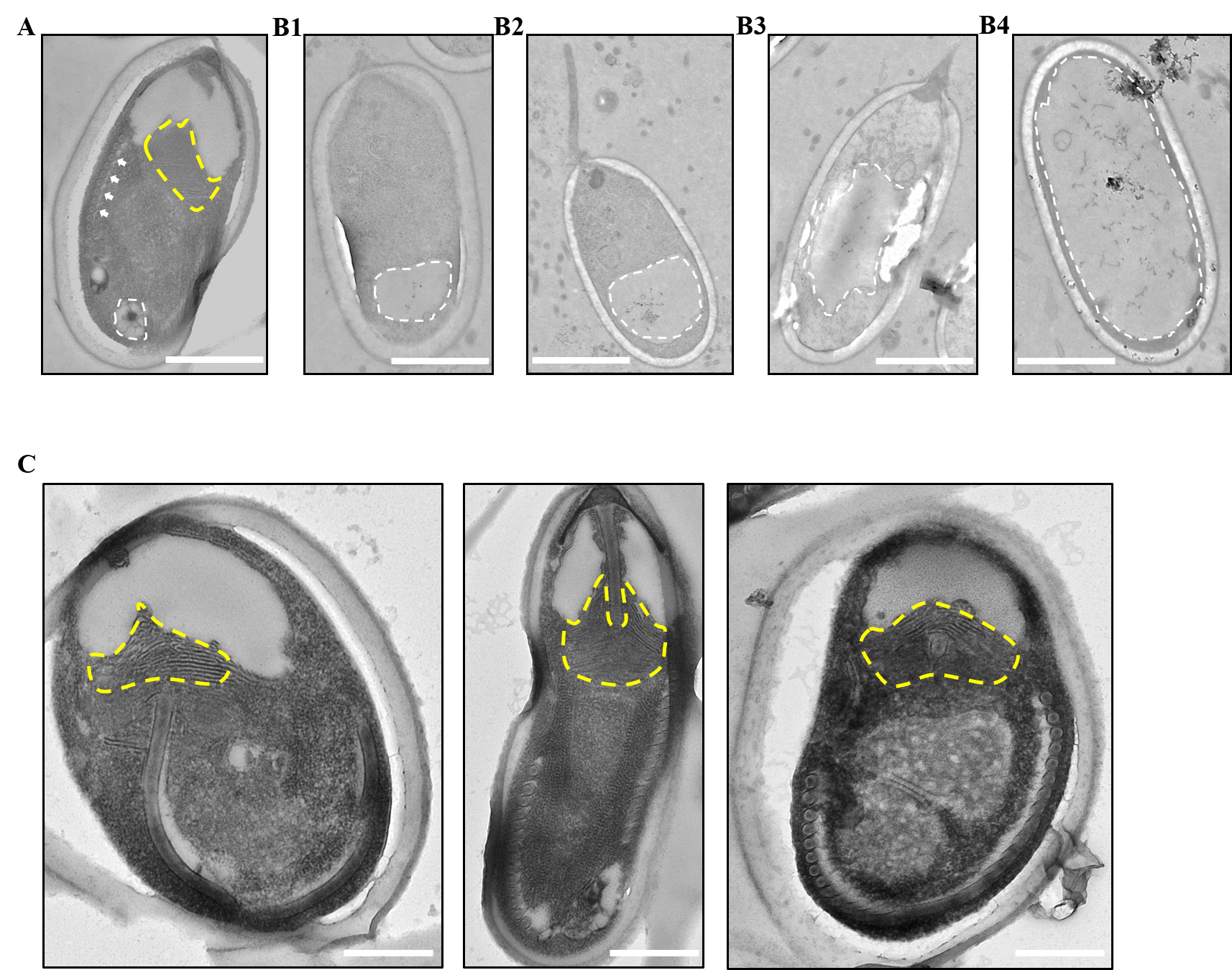

Supplement: Fig. S4 — TEM analysis of the ungerminated and germinated spores of N. bombycis. [file mbio.02749-23-s0004.tif]

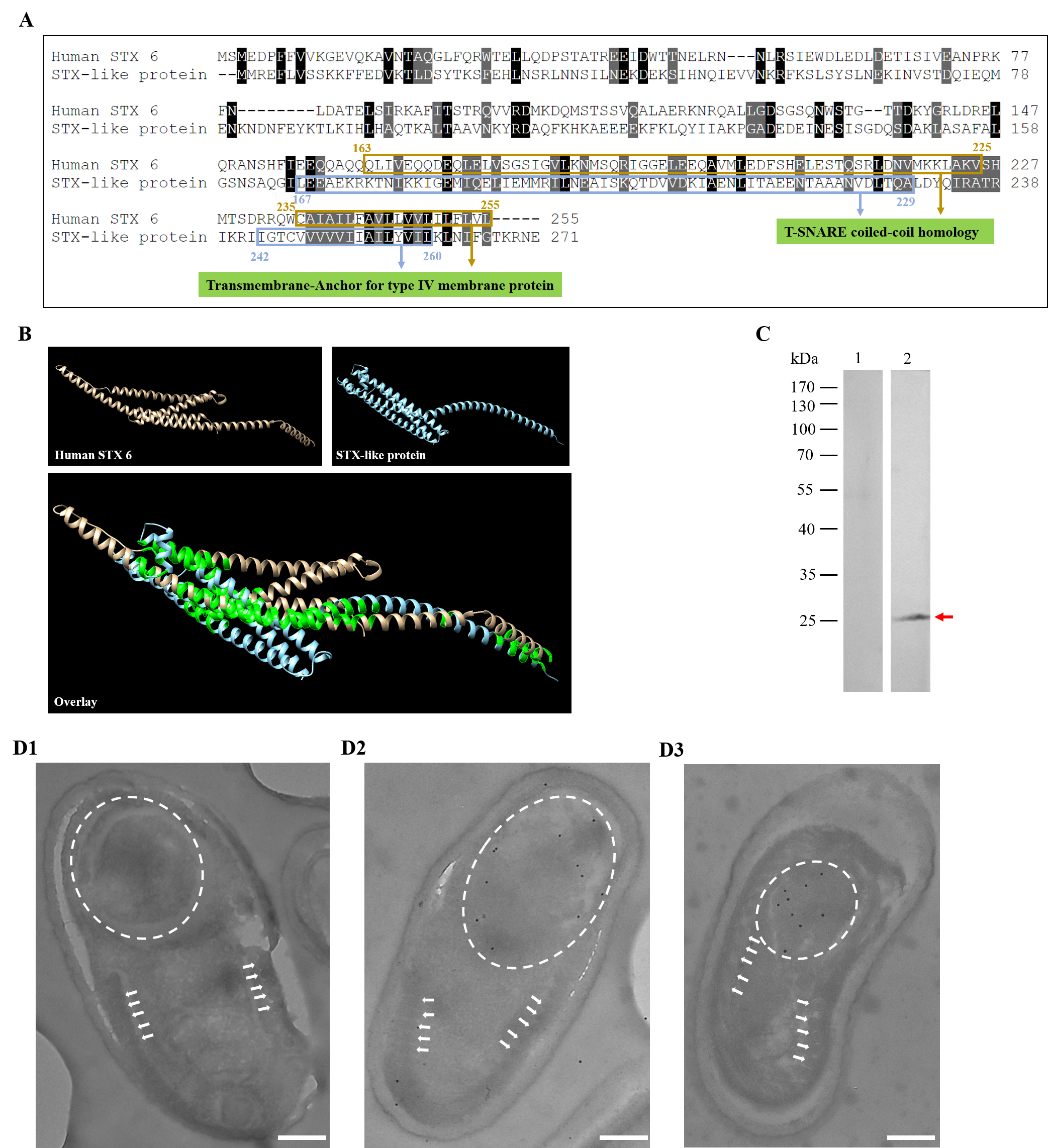

Supplement: Fig. S5 — Characterization of the STX-like protein in N. bombycis. [file mbio.02749-23-s0005.tif]
