# Supplementary material for: Microsporidia dressing up: the spore polaroplast transport through the polar tube and transformation into the sporoplasm membrane
Source: mBio. 2024 Jan 9;15(2):e02749-23. doi: 10.1128/mbio.02749-23 (PMC10865828; doi:10.1128/mbio.02749-23)
Supplement: Supplemental Information — Legends for Fig. S1 to S5. [file mbio.02749-23-s0006.docx]

**Supplementary Information**

**Fig. S1 TEM analysis of the extruded polar tube of *N. bombycis*.**

1. Spores were active to germinate by 0.1 M KOH on the grid, and the polar tubes (red triangles) were released. Bar, 10 µm. (B) The length of polar tubes was statistic analyzed. Each dot represented one polar tube (n=50). (C) TEM observation of the polar tube released from the mature spore. The green triangles in the enlarged image pointed the discontinuous high electron density materials in the polar tube. Bar, 5 µm or 1 µm (enlarged panels). (D) TEM analysis of the curved hook structure at the tip of the polar tube. The yellow triangles pointed the curved hook at the tip of polar tube. Bar, 500 nm. (E) Pie chart analysis of the proportion of polar tube with hook and without hook (n=100).

**Fig. S2 Purification of the polar tube and sporoplasm from *N. bombycis* and quantitative lipidomic analysis.**

1. Immunofluorescence assay of the purified polar tubes. The polar tubes were treated with rabbit anti-NbPTP1 serum (green). The nuclei were labeled with DAPI (blue). Bar, 10 μm. (B) Fluorescence assay of the purified sporoplasms. The nuclei were labeled with DAPI (blue) and the cell membrane of sporoplasms was labeled with DiI (red). Bar, 2 μm. (C) Pie chart analysis the lipid composition of polar tube and sporoplasm. Different lipid types were represented by different colors, and the proportion was represented by the size of the color block area. PC: Phosphatidylcholine; PS: Phosphatidylserine; PA: Phosphatidic acid; PE: Phosphatidyl ethanolamine; So: Sphingosine; PI: Phosphatidylinositol; DG: Diacylglycerol.

**Fig. S3 Structure characteristics of the polar filament in *N. bombycis*.**

(A) Immunofluorescence analysis of the purified polar filament fragments. The polar filaments were treated with rabbit anti-NbPTP1 serum (green). The nuclei were labeled with DAPI (blue), and the membrane structure was labeled with DiI (red). The white arrows referred to the polar filaments with relative long filament structure. Bar, 5 μm. (B) Cryo-EM observation the structure characteristics of the polar filament. Bar, 100 nm.

**Fig. S4 TEM analysis of the ungerminated and germinated spores of *N. bombycis*.** (A) The vertical section of ungerminated spores. The white arrows represented the polar filament, the white dotted line represented the region of PV, and the yellow dotted line represented the region of polaroplast. Bar, 1 μm. (B) The vertical sections of germinated spores. The white dotted line represented the region of PV. Bar, 1 μm. (C) The polaroplast in mature spores. The yellow dotted line represented the region of polaroplast. Bar, 500 nm.

**Fig. S5 Characterization of the STX-like protein in *N. bombycis*.**

(A) Sequence alignment analysis of human STX 6 (Genbank No. CAG46671.1) and *N. bombycis* STX-like protein (Genbank No. EOB15057.1). The golden and blue boxes represented the functional domains and transmembrane domains respectively. Identical and similar residues were highlighted in black and grey respectively. (B) AlphaFold models analysis for human STX 6 and *N. bombycis* STX-like protein. The overlapping region was highlighted by the green fluorescence. (C) Western blot analysis of STX 6 expressed in the total spore protein of *N. bombycis*. Lane 1: immunoblotting analysis of negative rabbit serum in the total spore protein. Lane 2: immunoblotting analysis of rabbit anti-STX 6 serum in the total spore protein. The red arrow marked the *N. bombycis* STX-like protein band. (D) IEM analysis the immunolocalization of the STX 6 in mature spores. D1: Negative control. D2 and D3: Mature spores, with gold particles-labeled STX 6 polyclonal antibody localized the polaroplast region. The white arrows represented the polar filament and the white line circled the polaroplast region. Bar, 500 nm.
